# A critical interpretive synthesis of the roles of midwives in health systems

**DOI:** 10.1186/s12961-020-00590-0

**Published:** 2020-07-08

**Authors:** Cristina A. Mattison, John N. Lavis, Michael G. Wilson, Eileen K. Hutton, Michelle L. Dion

**Affiliations:** 1 Department of Obstetrics and Gynecology, McMaster Midwifery Research Centre, 1280 Main St. West, HSC-4H26, Hamilton, ON L8S 4K1 Canada; 2 McMaster Health Forum, 1280 Main St West, MML-417, Hamilton, ON L8S 4L6 Canada; 3 Department of Political Science, McMaster University, 1280 Main St. West, KTH-533, Hamilton, ON L8S 4M4 Canada

**Keywords:** Midwifery, Political systems, Health systems, Critical interpretive synthesis, Sexual and reproductive health and rights

## Abstract

**Background:**

Midwives’ roles in sexual and reproductive health and rights continues to evolve. Understanding the profession’s role and how midwives can be integrated into health systems is essential in creating evidence-informed policies. Our objective was to develop a theoretical framework of how political system factors and health systems arrangements influence the roles of midwives within the health system.

**Methods:**

A critical interpretive synthesis was used to develop the theoretical framework. A range of electronic bibliographic databases (CINAHL, EMBASE, Global Health database, HealthSTAR, Health Systems Evidence, MEDLINE and Web of Science) was searched through to 14 May 2020 as were policy and health systems-related and midwifery organisation websites. A coding structure was created to guide the data extraction.

**Results:**

A total of 4533 unique documents were retrieved through electronic searches, of which 4132 were excluded using explicit criteria, leaving 401 potentially relevant records, in addition to the 29 records that were purposively sampled through grey literature. A total of 100 documents were included in the critical interpretive synthesis. The resulting theoretical framework identified the range of political and health system components that can work together to facilitate the integration of midwifery into health systems or act as barriers that restrict the roles of the profession.

**Conclusions:**

Any changes to the roles of midwives in health systems need to take into account the political system where decisions about their integration will be made as well as the nature of the health system in which they are being integrated. The theoretical framework, which can be thought of as a heuristic, identifies the core contextual factors that governments can use to best leverage their position when working to improve sexual and reproductive health and rights.

## Introduction

Midwives’ roles in sexual and reproductive health and rights (SRHR) continue to evolve and an understanding of the profession’s role in health systems is essential in creating evidence-informed policies. Countries across all income levels face challenges with providing high-quality SRHR and achieving effective coverage [1]. National or sub-national SRHR policies often do not include the midwifery workforce or account for the professions’ role in the provision of high-quality care [1]. The lack of conceptual clarity regarding the drivers of midwives’ roles within health systems, ranging from their regulation and scope of practice to their involvement in care, has resulted in significant variability both within and across countries on how the profession is integrated into health systems.

Research on midwifery care has demonstrated that the profession delivers high-quality SRHR services [1–3]. Care provided by midwives who are trained, licensed and regulated according to international standards is associated with improved health outcomes [3–7]. While midwifery care is associated with positive outcomes, it is an area that is under-researched [8]. This is particularly true in relation to how political and health system factors influence the profession’s role in health systems. As such, the roles of midwives in health systems are not clearly understood, which continues to challenge the profession’s ability to work effectively in collaborative and interprofessional settings.

Midwifery research is often dichotomised by the development status of the jurisdiction of focus — high-income countries (HICs) compared to low- and middle-income countries (LMICs). In HICs in general, midwives’ roles are focused on primary care to low-risk pregnant people through pregnancy, labour and a limited post-partum period [9]. In comparison, in LMICs, midwives’ scope of practice can be broader and extends to many aspects of SRHR [10–13]. International organisations (e.g. WHO, United Nations Population Fund and the International Confederation of Midwives) support an expanded approach to midwifery roles to include provision of a range of SRHR services (e.g. health counselling and education, prevention of mother-to-child HIV transmission, prevention and treatment of sexually transmitted infections, and provision of safe abortion where legal) [4, 14].

Arguably one of the most crucial components of a health system is its health workforce, as highlighted by WHO’s framework of ‘building blocks’ to support health systems strengthening (service delivery, health workforce, health information systems, access to essential medicines, financing and governance) [15]. While midwifery is recognised as key to SRHR, there is a global shortage of the midwifery workforce [2, 4]. Midwives who are educated and regulated according to international standards can provide 87% of a population’s essential SRHR, yet only 36% of the midwifery workforce is made up of such fully trained midwives, with a range of other health workers also delivering midwifery services [4]. The latter has been made possible by the range of roles that non-midwife health workers play in providing midwifery services [4, 16].

The lack of understanding of the roles of midwifery in health systems has led to significant disparities within and across countries. A better understanding of the roles of midwives within the health system is desirable as they are a key component in the delivery of safe and effective SRHR and could possibly improve the cost-effectiveness of the delivery of these services [17–19]. There is growing recognition that, to strengthen health systems, decisions must be based on the best available research evidence [20–23]. Using the available research evidence to understand the roles of midwives across health systems, as well as the political and health system drivers, will yield important insights with the aim of adding to the evidence base that policy-makers can draw from.

The present study asks — across health systems, what are the factors that influence the roles of midwives within the health system? We present a theoretical framework to explain how political and health system factors influence the roles of midwives within the health system. It defines the political system as consisting of three main components, namely institutions, interests and ideas [24]. ‘Health system arrangements’ are made up of governance, financial and delivery arrangements, and implementation strategies [25]. Given the lack of theoretical development in the area, this paper, through a critical interpretive synthesis of the available literature, identifies the factors that act as barriers or facilitators to the roles of midwives.

## Methods

### Design

A critical interpretive synthesis was used to develop the theoretical framework, which is an inductive approach to literature analysis. The approach uses conventional systematic review processes while incorporating qualitative inquiries to examine both the empirical and non-empirical literature [22]. Critical interpretive syntheses are best suited to developing theoretical frameworks that draw on a wide range of relevant sources and are particularly useful when there is a diverse body of literature that is not clearly defined, as is the case with literature related to the roles of midwives in health systems. Conventional systematic reviews have well formulated research questions at the outset, while a critical interpretive synthesis employs a compass question, which is highly iterative and responsive to the findings generated in the review process [26].

### Literature search

**Fig. 1:**
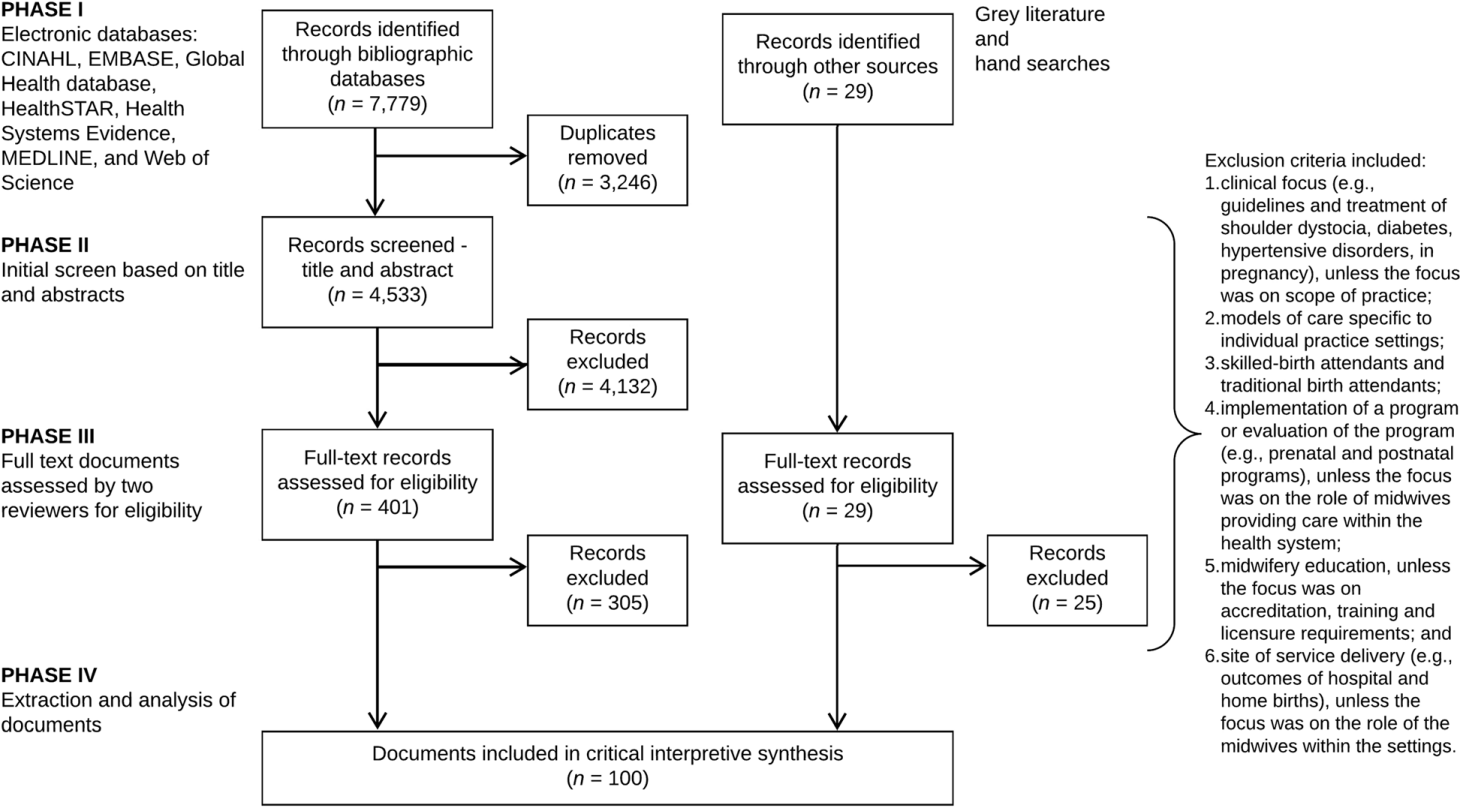
Literature search and study selection flow diagram

The selection of the literature was carried out in phases (Fig. 1). The first phase consisted of a systematic search of electronic bibliographic databases. The searches were executed in consultation with a librarian, who provided guidance on developing keywords (along with Boolean operators) and MeSH (Medical Subject Heading), refining the search strategy, identifying additional databases and executing the searches. We searched the following electronic databases through to 14 May 2020: CINAHL, EMBASE, Global Health database, HealthSTAR, Health Systems Evidence, MEDLINE and Web of Science. The search strategy was first developed in the MEDLINE database, using keywords and MeSH. Similar search strings were used across databases, with minor adjustments made to ensure search optimisation. The searches in MEDLINE included midwi* AND (roles OR scope), midwi* AND delivery of health care (MeSH), midwi* AND patient satisfaction (MeSH), midwi* AND quality of health care (MeSH), and midwi* AND standards (MeSH).

The second phase, complementary to the bibliographic database search, was a search of policy and health systems-related SRHR and midwifery organisation websites for relevant documents (e.g. World Health Assembly resolutions and United Nations Population Fund’s State of the World’s Midwifery reports). In addition, hand searches of reference lists from key publications were used to identify further relevant literature (e.g. 2014 *Lancet* Series on Midwifery). The final step in the literature search process was a purposive search to identify literature to fill the conceptual gaps that emerged.

### Article selection

For inclusion, the documents had to relate specifically to trained midwives, with leeway in terms of title (e.g. certified nurse-midwives and certified midwives in the United States). Articles were included, that in addition to providing insight into the compass question, also (1) incorporated a range of perspectives across different countries; (2) integrated different concepts into one document; and (3) included perspectives on the compass question from other disciplines (e.g. geographic information system and other techniques to map the distribution of the midwifery workforce). In order to incorporate a broad range of documents, there were no limits placed on the searches such as regarding language or publication year.

An explicit set of exclusion criteria were developed by the research team to remove the documents that were not relevant to the aims of the study and did not link to the compass question. Exclusion criteria included documents (1) with a clinical focus (e.g. clinical guidelines, pharmacology, diagnostics, devices, surgery and/or treatment of shoulder dystocia, diabetes, hypertensive disorders, in pregnancy), unless the focus was on scope of practice (e.g. midwives working in expanded scopes); (2) focused on models of care that were specific to individual practices or hospitals and included those that were related to health system approaches; (3) relating to unskilled workers providing SRHR (e.g. traditional birth attendants); (4) focused on implementation of a programme or evaluation of the programme (e.g. prenatal and postnatal programmes), unless the focus was on the roles of midwives providing care within the health system; (5) focused on midwifery education, unless the focus was on accreditation, training and licensure requirements; and (6) focused on site of service delivery (e.g. outcomes of hospital and home births), unless the focus was on the roles of midwives within the different practice settings.

Once the series of searches were completed, an Endnote database was created to store and manage the results. All the duplicates were removed from the database and an initial review of the titles and abstracts was performed for each entry by the principal investigator (CAM) and records were classified as ‘possibly include’ or ‘exclude’. In the first stage of screening, records were marked as ‘possibly include’ if they provided insight into the study’s compass question. Full-text copies of the remaining records were retrieved and uploaded to Covidence, an online tool for systematic reviews, for final screening [27].

The last stage of screening involved two phases and consisted of full-text review by three reviewers (CAM, TD and KMB). Using Covidence, each reviewer examined the records independently to assess inclusion. Any discrepancies were discussed and resolved. The reviewers prioritised the inclusion of empirical articles where possible, including empirical qualitative studies, which are the types of articles most likely to address political and health system components.

### Data analysis and synthesis

A coding structure was created to guide the data extraction. The areas of expertise of the authors (health systems and policy, clinical practice and political science) informed the selection of frameworks guiding the data extraction. The political system factors were informed through the 3i framework, which is a broad typology that recognises the complex interplay among institutions, interests, and ideas and provides a way of organising the many factors that can influence policy choices [24, 28–30]. Institutions are made up of government structures (e.g. federal versus unitary government), policy legacies (e.g. the roles of past policies) and policy networks (e.g. relationships between actors around a policy issue). Interests can include a range of actors who may face (concentrated or diffuse) benefits and costs with particular courses of action, whereas ideas relate to peoples’ beliefs (including those based on research evidence) and values.

‘Health system arrangements’ were informed through an established taxonomy developed by the McMaster Health Forum that includes (1) governance arrangements (e.g. policy authority, organisational authority and professional authority); (2) financial arrangements (e.g. how systems are financed and health professionals remunerated); (3) delivery arrangements (e.g. how care meets consumers’ needs, who provides the care and where it is provided); and (4) implementation strategy (consumer- or provider-targeted strategies) [25]. The components of the framework for quality maternal and newborn care (practice categories, organisation of care, values, philosophy and health professionals) were incorporated into the health system arrangements coding structure to yield insights specific to midwifery care [3].

In addition to the frameworks that guided data extraction, further data was collected on publication year, study design and jurisdiction(s) of focus. A data extraction form was developed based on all of the concepts covered in the frameworks as well as the additional descriptive items.

The critical interpretive synthesis was conducted on the high value articles — those that yielded the most insight into the compass question. The reviewers prioritised the inclusion of empirical articles that were conceptually rich or integrated different concepts, filled disciplinary gaps, captured a breadth of perspectives across different countries or applied approaches outside of health. The articles were read by the principal investigator (CAM) and one- or two-page detailed summaries were created for each article. The summaries were coded using the qualitative software NVivo for Mac, which facilitates the organisation and coding of the data [31]. Coding was informed by the three key frameworks guiding the analysis and outlined above: 3i framework, ‘health system arrangements’ and components of the framework for quality maternal and newborn care.

Three steps were involved in the analysis for the critical interpretive synthesis. First, the summaries of the articles were coded based on the coding structure outlined in the data extraction form. Using a constant comparative method, emerging data were compared to previously collected data to find similarities and differences [32, 33]. The approach included observations on the terms and concepts used to describe midwifery within the health system as well as relationships between the concepts. For example, how the role of midwives within the health system is influenced by policy legacies (i.e. institutions), which is related to problems with collaborative/interprofessional environments (i.e. delivery arrangements, skill mix and interprofessional teams). Second, all the data collected under each code was reviewed and more detailed notes of the concepts that emerged were included in the analysis. Lastly, themes were created for the concepts that emerged throughout the analysis.

Completeness of the findings was ensured through ongoing consultation with members of the research team. Central concepts and emerging themes of the study were discussed as a team and applied to current scholarship within the field of health systems and policy.

## Results

### Search results and article selection

A total of 7779 records were identified through the searches of electronic bibliographic databases. Once duplicates were removed (*n* = 3246), the remaining records (*n* = 4533) were screened based on title, abstract and the explicit set of exclusion criteria outlined above, leaving 401 potentially relevant records. In addition to the electronic database search, 29 records were purposively sampled for inclusion through grey literature and hand searches. The remaining 401 documents from the electronic database searches and 29 documents from the grey literature and hand searches were assessed by the reviewers (CAM, TD and KMB) for inclusion using the full text. A total of 100 documents were included in the critical interpretive synthesis (Fig. 1).

Over three-quarters (79%) of the documents were published after 2010, with no documents published prior to 2000. Of the 100 documents, the majority were primary research (*n* = 78), which were mostly qualitative research (*n* = 24) and observational studies (*n* = 24), followed by the ‘other’ category (*n* = 18) (e.g. geographic information systems research), systematic reviews (*n* = 15) and mixed methods (*n* = 4), while 1 was a randomised control trial. The remaining documents were categorised as non-research (*n* = 22), meaning that the approaches taken in the documents were either not systematic or that the methods were not reported transparently. Of the non-research documents, 8 were theoretical papers, 7 were reviews (non-systematic), 4 were ‘other’ (e.g. World Health Assembly resolutions, toolkits, etc.), and the remaining 3 were editorials. Forty-one of the documents focused on LMIC settings, followed by 35 on HIC settings, and 24 focused on both HIC and LMIC settings.

The results of the critical interpretive synthesis focused on the political and health system factors that influenced the roles of midwives within health systems. Table 1 focuses on the political system factors that emerged from the analysis and presents the relevant themes, relationships with other factors, and key examples from the literature of the factors that acted as either barriers or facilitators to the roles of midwives within the health system. Similarly, Table 2 focuses on the health system factors and presents the relevant themes, relationships with other factors, and key examples from the literature on the ‘health system arrangements’ that either acted as barriers or facilitators to the roles of midwives.

**Table 1:** Political system factors that influence the roles of midwives within the health system

Political system factors	Relevant themes	Relationships with other factors	Key examples from the literature		Sources
			Barriers	Facilitators	
Institutions					
Government structures	• Indigenous self-government allows communities to make decisions and implement midwifery services	• Variation in government structures can lead to differences in midwifery policy – relates to policy instruments (legislation and regulation)	•	• Self-government and political autonomy in Nunavik helped Inuulitsivik implement midwifery services during a time where midwifery was not a regulated profession (Canada) [34–36]	[34–39]
Policy legacies	• Past policies about the value of midwives creates interpretive effects, shaping the way midwifery care is organised in the health system • Values include SRHR policies that reinforce structural gender inequalities in a medical model, payment systems privileging physician-provided and hospital-based services	• Policy legacies ties closely to ideas as the values/mass opinion about ‘what ought to be’ are shaped by legacies of gender equality/inequality and vice versa	• Lack of professional recognition limited the establishment of midwifery (Bangladesh and Nepal) [40], consistent with gender inequality (Morocco) [41] and midwives faced gender discrimination and violence in the workplace [42–44] • Destruction of the health system as a result of conflict, which forbade education for women and resulted in a significant loss of the midwifery workforce (Afghanistan) [45, 46] and societal reconstruction post conflict (Cambodia) [13] • Policies in HICs that supported the medicalisation of birth, including hospital-based and physician-led care [47–53] • Historical prioritisation of training physicians over other health professionals [11, 54] • Loss of Indigenous midwifery as a result of colonisation and assimilation policies (e.g. evacuation of pregnant women out of the community and the residential school system) (Canada and Australia) [34, 54–58] • Caste system devalued midwifery because the profession is traditionally led by women caring for women (India) [59] • Midwives faced structural barriers to integration as a result of previous restrictive policies (e.g. midwives did not have a budget code in Mexico until 2011) [38, 60] • Lack of gender-sensitive and rights-based policies reinforced structural gender inequalities (i.e. created barriers to respectful maternity care and participation in policy-making) [42–44]	• Policy legacies that valued midwives and home births influenced the way the health system was organised (Netherlands) [49] • Midwifery as a tool to empower women and advance gender equality [61] • Professionalisation of midwifery began in the eighteenth century (Sweden) [62] • Universal Rights of Childbearing Women in the Respectful Maternity Care Charter, recognised that issues related to gender equity and gender violence were at the centre of maternity care – ‘safe motherhood’ extends to basic human rights for pregnant women [42, 63, 64] • The State of the World’s Midwifery 2014 was a global policy initiative that increased the status of midwifery at country levels and international policy dialogue [65, 66]	[11, 13, 34, 40–66]
Interests	• Interests include societal interest groups (e.g. consumer and religious groups), researchers, professional and international associations, and donor agencies • Policies are influenced by interests that have concentrated benefits and diffuse costs • Interest groups play a role in supporting or opposing the integration of midwifery in the health system • In LMICs, bilateral and multilateral donors work alongside local governments • In HICs, professional associations play a strong role in political lobbying	• Interests are closely related to institutions (policy networks) as well as ideas as interest groups often reflect and/or can influence societal values • Interest groups play an important role in advancing midwifery in the health system by (1) creating partnerships to improve SRHR [45, 67]; (2) promoting regulation and accreditation (e.g. accreditation requirements, setting standards, policies and guidelines) [63, 68–70]; (3) capacity-building, including midwifery research [71, 72]; (4) policy leadership and decision-making [43]; and (5) lobbying governments/advocacy [73, 74]	• Strong physician and hospital interest groups created a monopoly over maternity care (United States, Canada, Australia, and Mexico) [37, 38, 51, 55, 75–77] and impede midwives from practicing to their full scope [78, 79] • Tensions within the profession between nurse midwives and midwives (United States) [80] • Marginalisation of midwifery through dominant stakeholder groups [50] • Competing interests from nursing organisations created interprofessional tensions (Nepal) and limited establishing midwifery as an independent profession [81] • Barriers existed in accessing evidence published by African midwives (e.g. African nursing and midwifery research is often published in non-indexed journals) [72]	• Creation of interest groups to participate in the policy-making process [4] and strengthening existing groups in order to participate in the decision-making process (Nepal) [81, 82] • Consultations with interest groups to create culturally safe midwifery care (Canada) [34, 56–58] • Professional interest groups came together to strengthen health systems through (1) awareness campaigns; (2) lobbying (agenda-setting); and (3) training, advocacy and coalitions of interested stakeholders to inform education and policy [11, 66–68, 83] • Midwifery organisations used counter social movements to influence public opinion [49] • Researchers advocated for evidence-informed policies on midwifery [47] • Collaborative networks of health professional groups raised awareness of rising caesarean rates (Latin America) [84] • Professional associations and donor agencies advocated for scale-up and capacity-building of midwifery [61, 66, 73, 85] and supported local governments in the development of policies, regulatory activities, education and guidelines [11, 41, 45, 68, 69, 71, 86–89] • Strong leadership from midwifery professional associations engaged in policy dialogue and decision-making to advance universal health coverage and meeting health-related UN Sustainable Development Goals [8, 63, 66, 71, 90] • Equitable alliance between midwifery and physician groups (Sweden) [62] and collaborative professional development (United Kingdom) [76] • Increase in the number of midwifery professional associations in LMICs, which were enablers to advocacy and linking policy and implementation [87, 91] • Twinning (Tanzania Midwives Association and the Canadian Association of Midwives) strengthened midwifery professional associations and increased midwifery capacity [92] • Increase of research capacity by midwives supported teaching and clinical practice [72, 93]	[1, 4, 6, 8, 11, 12, 34, 35, 37, 38, 45, 47, 50, 51, 55, 57–59, 61, 63, 66, 67, 69, 70, 72, 73, 75–78, 80, 81, 83, 85–91, 93–98]
Ideas	• Societal values regarding gender equality (e.g. women’s roles within society) as well as the medical model (e.g. the medicalisation of the birth process and associated valuing of physician and hospital-based care)	• Ideas relate to both political and health system factors by influencing the values of citizens and either valuing or devaluing gender and the medical model	• Social construction of gender — the status of midwives in a given jurisdiction often reflected the value placed on women within the society (i.e. ‘gender penalty’) [8, 11, 41, 43, 46, 48, 61, 71] • Some cultures did not allow women to receive care from men yet there were few health professionals that were women due to lack of educational opportunities [45] • Health system priorities as well as changing values were based on the medical model and normalisation of medical interventions, which favoured care by physicians and within hospital settings [41, 48–50, 75, 78, 99–101] • Incongruence between international recommendations for skilled birth attendants and needs of Mayan population in Guatemala for intercultural healthcare from traditional birth attendants [102]	• Nordic maternity care systems’ non-medical models and women dominated professional groups [37]; respect of gender equality and informed choice [86] • Increasing consumer demand for midwifery-led care [77] • Reclaiming Indigenous midwifery and bringing birth back to the community (Canada and Guatemala) [35, 103]	[1, 3, 6–8, 10–13, 35, 37, 38, 41–43, 45–50, 54–58, 61, 62, 68, 71, 75, 77, 78, 84, 86, 94, 95, 97, 99, 100, 102–105]

*HICs* high-income countries, *LMICs* low- and middle-income countries, *SRHR* sexual and reproductive health and rights

**Table 2:** Health system arrangements that influence the roles of midwives within the health system

Health system arrangements	Relevant themes	Relationships with other factors	Key examples from the literature		Sources
			Barriers	Facilitators	
Governance arrangements	• Mechanisms to support accountability for state sector’s role in financing and delivery • The regulatory process (or lack thereof) of the profession is central to the roles of midwives within the health system and many references covered regulation as well as barriers to regulation • Accreditation systems to establish quality education • Enabling professional environments support the International Confederation of Midwives’ three pillars (education, regulation and professional association) • Scope of practice — expanding scope or restrictions to practicing to full scope	• Within governance arrangements, regulatory process overlaps with: • organisational authority – accreditation and • professional authority – training and licensure requirements, and scope of practice • Regulatory process overlaps with ‘ideas’ and in some cases self-regulation was a response to growing consumer demand for midwifery services [3]	• Lack of legislation to support regulatory activities [34, 43, 48, 58, 71, 82, 87, 93, 94] limited recognition and scope [38, 87] and the ability to practice as an autonomous profession [80] • Midwives lacked ownership and leadership to contribute to national accountability through tracking and reporting systems (e.g. midwives collecting or sharing data) [43, 65, 90] • Midwives were unable to practice to full scope because of inconsistent standards of education and professional regulation [78, 91, 106] • Globally, there was a general lack of knowledge regarding the International Confederation of Midwives’ Global Standards for Midwifery Education, which was a barrier to the provision of quality midwifery education [53, 66, 87, 107, 108] • Midwives were not practicing to their legislated full scope of practice (Canada), barriers included (1) hospitals — scope restrictions; (2) capping of the number of midwives granted hospital privileges; (3) capping the number of births attended by midwives; and (4) inconsistent midwifery policies across hospitals [52, 77] • Healthcare reforms increased the centralisation of decision-making, which created barriers to change (Australia) [95]	• Combination of regulatory processes and health systems that promoted birth as a natural process; favoured professional midwifery care (Nordic countries) [8, 62, 86, 91, 99] • Accreditation mechanisms supported midwifery education programmes and institutional capacities [63, 70, 93, 107] • Environments that allowed midwives to practice autonomously and to full scope of practice [74] • Expanded scope from providing skilled delivery care to include SRHR ranging from abortion, family planning, screening (diabetes and several forms of cancer), immunisations, palliative care, and public health and promotion [10–13, 55, 74, 94, 109–113] • Increased contraceptive prevalence rate (Nigeria) by engaging midwives in provision of family planning services [114] • Engagement of midwives within broader humanitarian emergency contexts (e.g. conflict, epidemics, and natural disasters) [46] • Effective collaboration between governmental institutions and professional associations supported quality midwifery education [107] • Integrated data collection and analysis into regional and national health information systems supported monitoring and evaluation processes for evidence-informed decisions [63]	[1–4, 6, 8, 10–13, 34, 35, 38, 39, 43, 45–48, 50, 52, 53, 55, 56, 58, 59, 62, 63, 65, 68–71, 74, 77, 78, 80, 82, 84, 86, 87, 90, 91, 93–100, 105–109, 111–116]
Financial arrangements					
Financing systems	• Financing systems: (1) Medicare has been funded by a mix of federal government cash payment to provinces, province- specific taxes and federal government (Canada) [6]; (2) mixed health system — public and private financing, health insurance, and service delivery and the public system is supported by the National Health Fund, which covers almost 75% of the population (Chile) [84]; and (3) effective coverage — the proportion of the population who need the intervention and receive it [1]	• Relates to ‘governance arrangements’ (accountability in the state sector’s roles in financing and delivery)	• Marginalisation of midwifery through reframing maternity care to focus on patient safety and costs of medical malpractice (United States) [50] and shifting of professional role boundaries between obstetricians and midwives [101] • Changes in the 1970s to the Canadian northern health services resulted in the evacuation of women from remote communities to hospitals in larger centres for childbirth [35] • Economic barriers to the provision of quality midwifery care included low or absent wages (e.g. waiting up to 6 months for public salary), lack of financing systems through governmental support, obligatory user fees and reimbursement by fee exemption schemes [43]	• Supportive policies were implemented through community-based and institutional healthcare services, which expanded across the country and were free (reaching most remote and rural areas) (Sri Lanka) [69] • The Government Midwifery Incentive Scheme, a nationwide results-based financing initiative increased (1) health system performance; (2) facility deliveries; and (3) skilled birth attendance (Cambodia) [115] • Incentivising facility deliveries through governmental initiatives to remunerate midwives and providing incentives to both the health professional and the client (Cambodia) [13, 115] • Maternity care reform enabled midwives to access Medicare and the Pharmaceutical Benefits Scheme (Australia)	[1, 2, 6, 10, 13, 35, 38, 39, 43, 50, 55–59, 61, 69, 73, 74, 76, 80, 84, 95, 101, 104, 109, 115]
Delivery arrangements	• The roles of midwives in health services delivery • Delivery arrangements relate to (1) access midwifery care (e.g. workforce supply, distribution and retention); (2) how care is provided (e.g. task-shifting, interprofessional teams); and (3) where care is provided (e.g. hospital based, integration of services and continuity of care)	• Delivery arrangements link with ‘institutions’, ‘interests’ and ‘ideas’ in that they influence the delivery of healthcare services	• Unmet need for SRHR services in sub-Saharan Africa due to health workforce supply and demographic trends [117] • Re-emergence of traditional midwives as a result of limited skilled birth attendant workforce [46] • Midwives experienced role strain due to increasing workloads [48], burn out [43, 118] and lack of support to practice autonomously [75, 104] leads to disempowerment [43] • Lack of equipment in schools and facilities can create gaps in teaching quality and practice [119] • Medical model prioritised physician-led care in hospitals and created friction between midwives and physicians [38, 50, 52] and also minimised the roles of midwives in primary care [99] • When compared with eight HICs, midwifery in Canada played a relatively minor role in the provision of SRHR [6] • Rising caesarean rates in Latin America and medically induced labours [84]	• Collaborative care involved interprofessional groups (e.g. midwives working with physicians and nurses) [10, 34, 55, 74, 75, 100, 116] • Based on statistical modelling, the projected effect of scaling-up midwifery will deliver the most impact on maternal, newborn and child health [2, 73] • Task-sharing of HIV, tuberculosis [96], abortion-related (medication abortion and vacuum aspiration abortions) services to midwives [12, 110, 111, 120] • Midwifery (led by Indigenous midwives) is returning culturally safe and appropriate SRHR to Inuit communities (Canada) [34–36, 54] • Midwives increased access to SRHR services in fragile and conflict-affected states [121, 122]	[3, 4, 6, 7, 9–13, 34–39, 43, 45, 46, 48, 50, 51, 54, 55, 58, 59, 61, 62, 69, 73, 74, 76, 77, 79, 86, 94, 96, 97, 99, 100, 104, 105, 110, 116–118, 120–122]

*HICs* high-income countries, *SRHR* sexual and reproductive health and rights

Three main findings emerged from the analysis on political system factors. First, within institutions, the effects of past policies regarding the value of midwives created interpretive effects, shaping the way midwifery care is organised in the health system. The legacies of these policies created barriers, which include SRHR policies that reinforced structural gender inequalities as well as, in a medical model, payment systems privileging physician-provided and hospital-based services [11, 13, 34, 41–45, 47–52, 54–59, 61–63, 65].

Second, interest groups played an important role in either supporting or opposing the integration of midwifery in the health system. These groups can have direct or indirect influence and policies that provide concentrated benefits and diffuse costs for groups are more likely to move forward [24]. Interest groups advanced the integration of midwifery in the health system by (1) creating partnerships to improve SRHR [45, 67]; (2) promoting regulation and accreditation (e.g. accreditation requirements, setting standards, policies and guidelines) [63, 68–70]; (3) capacity-building including midwifery research [71, 72]; (4) policy leadership and decision-making [43]; and (5) lobbying governments and advocacy [73, 74]. Strong leadership from midwifery professional associations engaged in policy dialogue and decision-making has helped advance agendas related to universal health coverage and meeting health-related United Nations Sustainable Development Goals [8, 63, 66, 71, 90].

Third, the most relevant themes related to ideas that emerged from the analysis pertained to societal values regarding gender (women’s roles within society) as well as the medical model (historical medicalisation of the birth process and associated growth of physician-provided and hospital-based care). We recognise the importance of gender-inclusive language but have use the term ‘women’ in this publication to reflect how gender is referenced in the documents reviewed. Barriers created by societal values included (1) social construction of gender and the status of midwives in a given jurisdiction often reflected the value placed on women within society (i.e. ‘gender penalty’) [8, 11, 41, 43, 46, 48, 61, 71]; (2) some cultures and beliefs did not allow women to receive care from men, yet there were few health professionals who were women due to lack of educational opportunities and societal values that restrict women from participating in the paid labour force [45]; and (3) health system priorities and shifting societal values favoured the medical model [41, 48–50, 75, 78, 99–101]. Examples of facilitators included Nordic health systems that value non-medical models and women-dominated professional groups [37], which respect the right to informed choice [86].

Within health system factors, the main themes that emerged from the literature are presented according to ‘health system arrangements’. First, within governance arrangements, regulation and accreditation mechanisms to support midwifery education programmes and institutional capacities were central to how midwives are integrated into health systems [63, 70, 93, 107]. The lack of legislation to support regulatory activities [34, 43, 48, 58, 71, 82, 87, 93, 94] limited recognition and scope [38, 87] and the ability for midwives to practice as an autonomous profession [80]. Globally, there was a general lack of knowledge regarding the International Confederation of Midwives’ Global Standards for Midwifery Education, which was a barrier to the provision of quality midwifery education [53, 66, 87, 107, 108]. Within financial arrangements, the literature focused primarily on how systems are financed, on the inclusion of midwifery services within financing systems and on the remuneration of midwives that is reflective of scope of practice [1, 2, 6, 10, 13, 35, 38, 39, 43, 50, 55–59, 61, 69, 73, 74, 76, 80, 84, 95, 101, 104, 109, 115]. Lastly, the main themes relating to delivery arrangements focused on (1) accessing midwifery care ranging from availability and timely access to workforce supply, distribution and retention; (2) by whom care is provided (e.g. task-sharing and interprofessional teams); and (3) where care is provided (e.g. hospital-based, integration of services and continuity of care) [3, 4, 6, 7, 9–13, 34–39, 43, 45, 46, 48, 50, 51, 54, 55, 58, 59, 61, 62, 69, 73, 74, 76, 77, 79, 86, 94, 96, 97, 99, 100, 104, 105, 110, 116–118, 120–122].

#### Theoretical framework

**Fig. 2:**
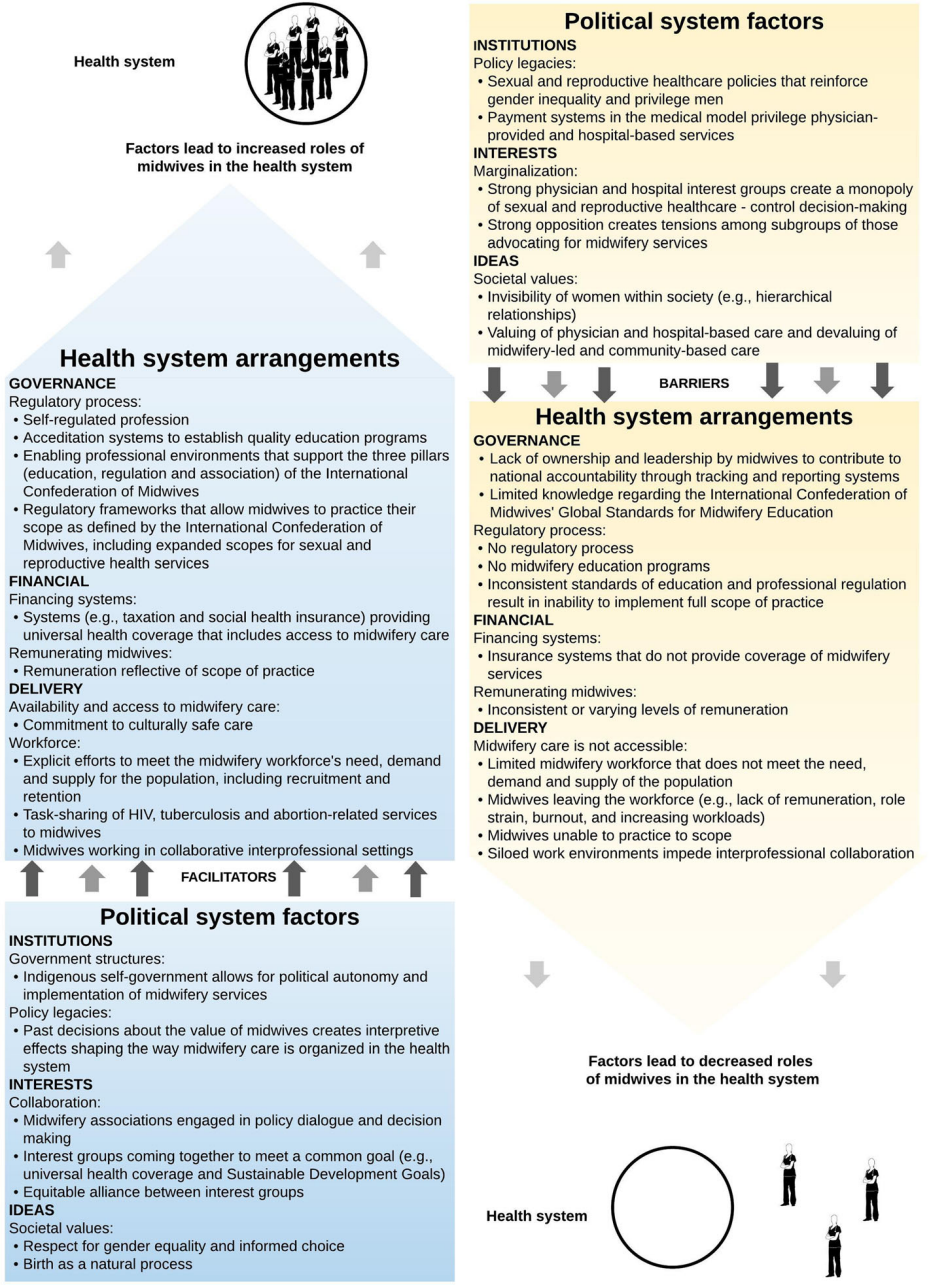
Theoretical framework of the political and health system factors that influence the roles of midwives within the health system

Figure 2 brings together the main findings from the critical interpretive synthesis and presents a theoretical framework, which can be thought of as a heuristic that can be used to map the key elements that influence midwives’ roles in a particular political and health system. The factors presented in the framework are not weighted but rather present the range of variables influencing the level of integration of the profession. The cumulative effects of the barriers presented on the right-hand side of the framework lead to health systems where the profession is disempowered and midwives exist on the margins with very limited capacity. Some of the variables and examples presented in the framework have context specificity to reflect findings from the critical interpretive synthesis (e.g. self-regulated profession, Indigenous self-government, Nordic maternity care systems, and payment systems privileging physician-provided and hospital-based services in some contexts).

## Discussion

### Principal findings

Similar to the concept of WHO’s health system ‘building blocks’, the political system factors presented in the theoretical framework form the bottom building block or the foundation for the ‘health system arrangements’, acting as either a barrier or facilitator. For example, favourable institutional factors (e.g. policy legacies that value midwifery as a profession), interests (e.g. collaborative interest groups coming together to reach a common goal) and ideas (e.g. societal values centring on gender equality and birth as a natural process) act as enablers to ‘health system arrangements’ that build on each other to support the integration of midwifery. Together, supportive political and health system factors lead to health systems where midwives practice to scope (i.e. trained, licensed and regulated according to international standards, working in collaborative/interprofessional settings with an established workforce). On the other hand, health systems that have many political and health system challenges will in turn have a limited midwifery workforce where midwives lack an institutional voice and representation in SRHR decision-making. Significant barriers limit the options available to the midwifery workforce and is most often reflected in siloed work settings with midwives working in the periphery of the health system.

### Strengths and limitations of the study

The main strength of the study is the use of a critical interpretive synthesis. This is a relatively new systematic review methodology, which combines a rigorous systematic review of electronic bibliographic databases with iterative and purposive sampling of the literature to fill conceptual gaps. The approach incorporated a range of documents (empirical and non-empirical), which broadened the scope of the literature used to inform the theoretical framework.

The main limitation of the critical interpretive synthesis was that the search strategy may not have fully covered the diverse terminology used to refer to midwifery. However, the principal investigator (CAM) consulted with a librarian and team members to ensure that the search strategy was as inclusive as possible, which is also reflected by the high proportion of articles that were later excluded during the screening process. Meanwhile, the majority of articles retrieved from the searches were published after 2000, which could be related to the release of the Millennium Development Goals and subsequent Sustainable Development Goals, and the wider attention given to SRHR on global agendas.

### Implications for policy and practice

Any changes to the roles of midwifery in health systems needs to take into account the political system where decisions about their integration will be made as well as the nature of the health system in which they are being integrated. The theoretical framework is a tool that helps to inform such changes by identifying the drivers of midwives’ roles that facilitate or constrain such integration. The study results have implications for policy-makers as, firstly, the theoretical framework can be used to conduct an assessment of the factors in order to strengthen the profession by identifying the facilitators that can be leveraged as well as the barriers that can be addressed to support change. For example, Sweden has favourable political system conditions (e.g. policy legacies of professionalisation of midwives dating back to the eighteenth century and an equitable alliance between midwifery and physician groups), which is reflected in the health system arrangements where midwives are the primary health professionals for low-risk pregnant people. In contrast, the United States has policy legacies of payment systems valuing physician-provided and hospital-based care, strong physician and hospital interest groups have created a monopoly over sexual and reproductive health services, and existing tensions within the profession between nurse midwives and midwives.

Moving forward, an implication for practice is that changes to further enhance the role of midwives would require different types of policy levers. In looking at growing midwifery in LMICs, governments can use the tool to understand how to best influence the integration of the profession. This information will provide valuable experience and understanding of the contextual factors so that governments can best leverage their position when working with bilateral and multilateral funders to improve SRHR. Conversely, in the example of the United States, the framework presented helps to explain why midwives play such a small role in sexual and reproductive health service delivery in the United States. The tool highlights that funding and regulatory levers would need to be pulled; yet, strong policy legacies and entrenched interests present significant barriers. Change would require spending political capital to modify existing structures within the health system.

## Conclusions

While research evidence on the role of midwives in the provision of high-quality SRHR has increased and the 2014 *Lancet* Series on Midwifery was key to raising the profile of midwifery research, significant gaps in the literature persist. Structural gender inequalities are reflected in the low status of midwifery in some contexts, which leads to poor political and health systems supports to invest in quality midwifery care [43]. Our findings show that the research evidence related to the roles of midwives within health systems is relatively saturated in terms of delivery arrangements yet surprisingly little is known about governance and financial arrangements and about implementation strategies, which are key to effectively integrating midwifery and pushing the field forward in meaningful ways.

## Data Availability

All data generated or analysed during this study are included in this published article and summarised in Tables 1 and 2.

## Abbreviations

HICs: High-income countries
LMICs: Low- and middle-income countries
SRHR: Sexual and reproductive health and rights
